# Preliminary Investigation of Fruit Mash Inoculation with Pure Yeast Cultures: A Case of Volatile Profile of Industrial-Scale Plum Distillates

**DOI:** 10.3390/foods13121955

**Published:** 2024-06-20

**Authors:** Josef Balák, Lucie Drábová, Vojtěch Ilko, Dominik Maršík, Irena Jarošová Kolouchová

**Affiliations:** 1 Department of Biotechnology, University of Chemistry and Technology, 6 Technická 5, 166 28 Prague, Czech Republic; josef.balak@vscht.cz (J.B.); dominik.marsik@vscht.cz (D.M.); 2 Department of of Food Analysis and Nutrition, University of Chemistry and Technology, 6 Technická 5, 166 28 Prague, Czech Republic; lucie.drabova@vscht.cz (L.D.); vojtech.ilko@vscht.cz (V.I.)

**Keywords:** volatile compounds, plum spirit, sensory characteristics, distillation, fermentation

## Abstract

This study investigates the effect of pure yeast culture fermentation versus spontaneous fermentation on the volatile compound profile of industrially produced plum brandy. Using traditional distillation methods, the evolution of key volatile compounds is monitored at seven different moments during the distillation process. By integrating advanced analytical techniques such as GC-MS and sensory evaluation, significant differences in the composition of the distillates are highlighted, particularly in terms of ethyl esters and higher alcohols which are key to the sensory properties of the final product. Distillates produced with the addition of pure cultures gave higher concentrations of esters than those obtained by wild fermentation. The results of our industrial research show that the most critical step is to limit the storage of the input raw material, thereby reducing the subsequent risk of producing higher concentrations of 1-propanol. Furthermore, our results indicate that the heart of the distillate can only be removed up to an ethanol content of approximately 450 g/L and that the removal of additional ethanol results in only a 10% increase in the total volume of the distillate, which in turn results in an increase in boiler heating costs of approximately 30%.

## 1. Introduction

Fruit distillates are alcoholic beverages, the production and consumption of which have a long tradition in countries all over the world. Europe, in particular, has a long tradition of producing fruit spirits. Germany, for instance, has over 15,000 distilleries that collectively produce nearly 4 million litres of ethanol annually [1]. In the Czech Republic, there are 64 large distilleries engaged in the production of fruit distillates with an annual output of approximately 12,000 hLe (hL of 100% ethanol). In addition, there are over 500 small distilleries with production volumes ranging from 20,000 to 40,000 hLe, depending on the fruit production in a given year. The quality and sensory acceptability of distillates are a direct consequence of the chemical composition of the distillate and can be influenced by the way each step of the production technology is performed. The better the raw material, the better the product [2,3,4,5]. The distillation process can only be controlled to a limited extent on an industrial scale. This is primarily achieved by controlling the intensity of heating, the rate of deflagration, and the choice of the time period when the so-called “heart” fraction is taken and used for the production of the spirit [1,2,6]. In addition to ethanol production, a number of other biochemical processes occur during fermentation, which is a process mediated by yeasts and other microorganisms present in the fermentation process [2,3,7]. The spectrum of compounds produced by yeast is contingent upon the composition of the mash, the conditions of the fermentation process, and the specific yeast species and strain [8,9]. The formation of methanol by demethylation of pectin occurs during fermentation but also during the entire storage period of fermented plum mash [10,11,12,13] until it is distilled [14]; therefore, plum ferment should not be stored for a long time [15], but this is not always possible due to capacity/operational reasons. Methanol is present in relatively high concentrations in the distillate and is released as a result of the breakdown of pectin by pectinases produced by yeasts, as well as by other microorganisms [16]. Methanol and its metabolites present toxicity risks, prompting regulatory limits on their concentration (Regulation (EU) 2019/787 of the European Parliament and of the Council) [17]. Acetaldehyde is the most significant carbonyl compound produced by yeast during fermentation [18]. The oxidation of acetaldehyde catalysed by yeast or bacteria produces acetic acid whose concentration depends on the concentration of this aldehyde [7]. Ethanol is the most abundant alcohol produced during fermentation, while ethyl acetate is the ester produced at the highest rate. The amino acids content of the original raw material has been demonstrated to influence the profile of higher alcohols [19,20,21]. The synthesis of ethyl esters of fatty acids is associated with the esterase activity of yeast. The rate of their production is contingent upon the respective yeast strain and the concentration of the precursors, acyl-CoA and ethanol. Ethyl esters represent the largest group of aromatic compounds in plum brandy. They are formed by yeast metabolism during fermentation and significantly influence the sensory character of the distillate [21]. Esters affect the overall profile of distillates more than higher alcohols, due to their low sensory perception thresholds [22].

Plums are a very popular fruit crop, especially in Europe. In terms of area, plum orchards are surpassed only by apple orchards [23]. A large part of plum production is processing in the distillery industry [24]. During the distillation of fruit ferments, it is crucial to identify an optimal balance between the ethanol yield, low content of toxic substances, and harmonious content of sensory active substances in the final product. The duration of the distillation process, during which the individual components of fruit sludge pass into the distillate, is contingent upon the interplay between physico-chemical properties of these substances and the chemical composition of the distilled mixture, which undergoes a transformation during distillation [6]. According to some authors, substances can be categorized by their sensory characteristics brought to the distillate—such as alcohols, carbonyl compounds, organic acids, esters, terpene compounds, lactones, phenols, alkanes, and others [4,25,26]—and by the distillation time at which they are distilled [7,21,27,28,29].

Substances with a low boiling point and high solubility in ethanol are distilled at the beginning of each distillation (e.g., acetaldehyde, ethyl acetate, and ethyl propionate). Compounds with a relatively higher boiling point, well soluble in ethanol, are distilled from the beginning to approximately halfway through the distillation process (e.g., ethyl hexanoate, ethyl octanoate, ethyl decanoate, ethyl dodecanoate, and isoamyl acetate). Compounds with a relatively low boiling point (not exceeding 200 °C), which are soluble in both ethanol and water, are distilled from the beginning of the distillation, and their concentration gradually decreases during the distillation (e.g., alcohol, 2-methylbutanol, and isoamyl alcohol). Compounds with a boiling point higher than the boiling point of water, which are soluble in water, are distilled from the middle to the end of the distillation process (e.g., acetic acid, 2-phenylethanol, ethyl lactate, and diethyl succinate). The concentration of the compounds resulting from chemical reactions during distillation, which have a high boiling point and are highly soluble in water, gradually increases from approximately halfway to the end of the distillation process (e.g., furfural) [4,7,21,22,23,24,25,26,27,28,29]. Because of the limited ability to control the distillation process and the legal regulations governing distillation and alcohol handling, it is not possible to manage the control over the distillates at the appropriate time.

The combination of individual chemical components forms a distinctive mixture with unique sensory properties within the complex matrix of fruit distillates. Some substances present in the distillate in very low concentrations significantly influence the organoleptic properties of fruit distillates, often through their synergistic effects [30,31,32,33,34]. It is noteworthy that distillates derived from disparate fruit types exhibit greater analytical and sensorial divergence than those derived from distinct varieties [26,35]. Although there are more than 170 varieties of plums [36,37], their distillates are not sold in a varietal distinction. Moreover, the economic benefit, in terms of turnover from its sale, is considerable [1,38]. The current trend in professional publications is towards the search for authentication features specific to a given fruit distillate, employing the latest analytical procedures [23,31,39]. Concurrently, these studies frequently examine the impact of various factors on the analytical and/or sensory characteristics of the distilled beverage, solely from the perspective of laboratory experiments [15,23,31,37,38,40,41,42]. For the practical application of these findings in industry, there is a lack of studies that investigate real operating conditions and volumes of raw input materials. This would allow manufacturers to improve the output product based on simple steps and analyses on available instruments.

A significant proportion of distilleries continue to employ the technique of spontaneous fermentation in the production of fruit spirits. This study examined the impact of incorporating a pure yeast culture on the profile of volatile compounds, compared to spontaneous fermentation on an industrial scale. Additionally, changes in the concentration of volatile compounds were monitored at seven time points during the collection of the “heart” of the distillate.

## 2. Materials and Methods

### 2.1. Samples of Plum Brandy

**Figure 1:**
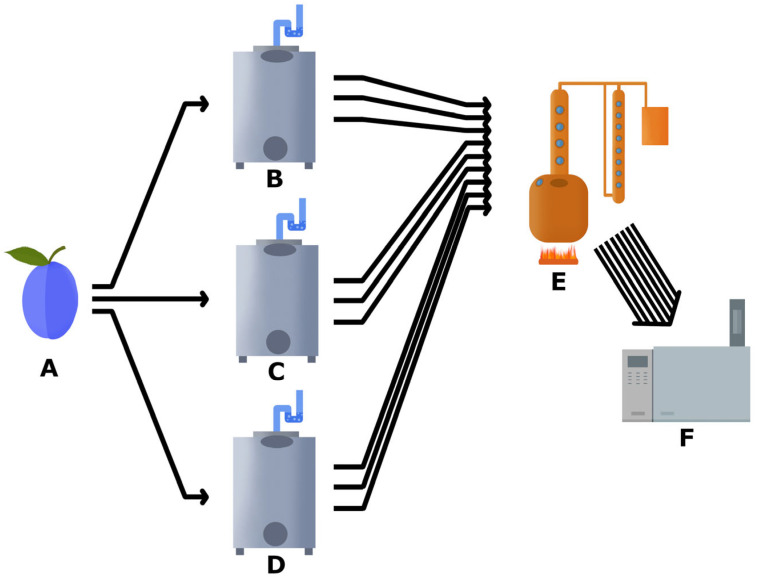
(**A**)—Raw material (plums) was disintegrated and split into three tanks. (**B**)—Fermentation provided by wild microflora. (**C**)—Fermentation with the addition of a pure yeast culture of strain SP7. (**D**)—Fermentation with the addition of a pure yeast culture of strain DV10. (**E**)—Distillation in a single pot still with column. (**F**)—Chemical analysis (GC-FID).

The distillates were derived from plums cultivated in the Moravia Region of the Czech Republic under comparable climatic conditions. The fermentation process was conducted in a 4 t vertical stainless steel tank containing 3.5 t of plums which had been disintegrated by a toothed roller crusher and without crushing stones. The alcoholic fermentation process was conducted in closed vessels at a temperature of 20 ± 2 °C under anaerobic fermentation conditions within an air-conditioned fermentation hall (Figure 1).

The fermentation process was conducted by the natural autochthonous microorganisms or with the addition of pure yeast cultures SP7 and DV10 (SAS Sofralab, Magenta, France). The SP7 yeast is designed for the fermentation of high concentrations of carbohydrates, while the DV10 yeast is exhibits a high tolerance to ethanol.

The selected yeast strains were chosen on the basis of their specifications and the problems encountered when fermenting plum kvass on a production scale, such as the residual content of fermentable carbohydrates. DV10 yeast is able to ferment over a wide temperature range (10–35 °C), has a low nitrogen concentration, a high tolerance to alcohol, and produces little foam. It is able to ferment under stressful conditions of low pH and low temperature. It is characterised by respecting the varietal character. It is characterised as *Saccharomyces cerevisiae bayanus*. SP7 yeast is able to ferment over a wide temperature range (10–32 °C), has a low nitrogen concentration, low volatile acid production, and produces little foam. It is able to ferment under stressful conditions of low pH and low turbidity. It produces elegant aromas and harmonious flavours.

The yeast was inoculated at a rate of 25 g/hL of yeast. The duration of the alcoholic fermentation process was two months. All plum varieties underwent distillation in a single pot still with a distillation column (volume 600 L), heated by a gas burner, one month after the conclusion of fermentation. Samples of the distillate were taken at seven distinct time points during the distillation process. Time 0 represented the beginning of the heart of the distillate, and other time periods were 5, 15, 30, 60, 120, and 180 min. The sampling intervals at the beginning of the collection of the “heart” fraction were shorter because the concentrations of the most volatile substances tend to change rapidly in the early stages of distillation. The “heart” fraction was collected when the ethanol content reached 50% (*v*/*v*). Summary distillate samples were taken as average samples from the collected distillate and standardised to 40% (*v*/*v*).

### 2.2. Chemicals and Reagents

All reference standards of methyl acetate, ethyl acetate, ethyl butyrate, octyl acetate, ethyl benzoate, propyl benzoate, hexyl hexanoate, isoamyl benzoate, isoamyl decanoate, ethyl hexanoate, 2-phenylethyl octanoate, methyl cinnamate, ethyl cinnamate, ethyl undecano-ate, ethyl (E)-2-decenoate, ethyl myristate, methyl palmitate, ethyl oleate, ethyl linoleate, ethyl salicylate, diethyl succinate, methanol, ethanol, 1-propanol, isopropanol, 1-butanol, 2-butanol, isobutyl alcohol, 2-methyl butanol, isoamyl alcohol, 1-pentanol, 1-hexanol, 1-heptanol, 1-octanol, 1-nonanol, 1-decanol, benzyl alcohol, 2-phenylethanol, 3-ethoxypropionaldehyde diethyl acetal, diethyl acetal, eugenol, geraniol, linalool, cit-ronellol, α-terpineol, acetone, hexanal, heptanal, farnesene, nonanal, isovaleraldehyde, furfural, benzaldehyde, 2,4-butanedione, damascenone, and ethyl carbamate were procured from Sigma-Aldrich Ltd. (Saint Louis, MO, USA). The purity of all individual standards was equal to or higher than 98%. Water purified by a Milli-Q^®^ Integral system supplied by Merck (Darmstadt, Germany) was used throughout the study.

### 2.3. Determination of Aroma Substances 

The identification of the distillate components was conducted using a gas chromatograph Agilent 6890N coupled to a 5975 mass spectrometer (GC-MS, Agilent Technologies, Palo Alto, CA, USA). For the separation of the target analytes, an HP-INNOWAX column (30 m × 0.250 mm i.d., 0.25 μm film thickness) obtained from Agilent Technologies (USA) was used. The GC conditions were as follows: oven temperature program: 40 °C (3 min); 5 °C min^−1^ to 240 °C (5 min); carrier gas helium with a flow rate of 1 mL min^−1^; injection mode: split (50:1); injection volume: 1 μL; and inlet temperature: 250 °C. The MSD interface temperature was set at 230 °C; quadrupole temperature at 150 °C; and ion source temperature at 230 °C. The mass spectrometer was operated in the selected ion-monitoring mode detecting at least two ions per analyte. Quantification of individual compounds was carried out using an Agilent 7890B gas chromatograph coupled to a flame ionisation detector (GC-FID, Agilent Technologies, Palo Alto, CA, USA). Target analytes were separated using an HP-INNOWAX column (60 m × 0.250 mm i.d., 0.25 μm film thickness) under the following conditions: 35 °C (8 min); and 5 °C min^−1^ to 250 °C (5 min). The carrier gas was nitrogen with a flow rate of 1.7 mL min^−1^; injection mode: split 1:50; and the injection volume was 1 μL. Analyses were carried out in triplicate and their averages were used as a single data point. Calibration was performed and repeated with a standard solution according Spaho et al. [21].

### 2.4. Sensory Evaluation

The results of the chemical analyses were complemented with a sensory analysis. The assessment of the fruit distillate of slivovitz was conducted at the Sensory Laboratory of the University of Chemistry and Technology Prague, which is equipped according to the ISO 8589 standard [43]. The assessors were meticulously selected, trained, and monitored in accordance with the international standards ISO 8586 [44], ISO 5496 [45], and ISO 3972 [46]. The triangle test was carried out in accordance with the ISO 4120 [47] standard. The aim of this test was to demonstrate a perceptible sensory difference between two different samples using the forced selection method. The evaluators received three samples, with one being different from the other two. They were asked to use their sense of smell and taste to identify the different sample and to indicate whether they found it easy or difficult to recognize. Those who correctly identified the different sample then moved on to pairwise comparative testing using the same samples from the triangular test. The pairwise comparison test was carried out in accordance with ISO 5495 [48]. A total of 39 assessors participated in the evaluation.

The profile test, as per ISO 13299 [49], engaged 20 trained assessors. Utilizing a 100-point unstructured scale, the assessment encompassed 24 descriptors covering the odour, taste, and flavour of the samples. The training also included several selected descriptors. All selected substances were evaluated in water. For the descriptor “intensity of fruity taste” and “intensity of fruity aroma”, samples with the addition of ethyl (E,Z)-2,4-decadienoate with a pear aroma were prepared at concentrations of 5, 10, and 20 mg/L. For the descriptor “intensity of odour”, samples with limonene at concentrations of 15, 20, and 50 μg/L were used. For the descriptor “intensity of vegetal odour”, samples with the addition of hexan-1-ol at concentrations of 30, 60, and 100 mg/L were prepared. For the descriptor “intensity of spicy odour”, samples with guaiacol at concentrations of 3, 5, and 8 μg/L were prepared. For the descriptor “intensity of resinous odour”, samples with α-pinene at concentrations of 1, 3, and 5 mg/L were prepared. For the descriptor “intensity of bitter almond odour”, samples with benzaldehyde at concentrations of 400, 600, and 800 mg/L were prepared. For the descriptor “intensity of technical (chemical) odour”, samples with the addition of methyl salicylate at concentrations of 1, 3, and 5 μg/L were prepared. The data collection and statistical processing of the sensory analysis were facilitated by the use of RedJade software (RedJade Sensory Solutions, LLC, Martinez, CA, USA).

### 2.5. Statistical Analysis

Statistical analysis was performed using MetaboAnalyst 6.0 software, where one-way Analysis of Variance (ANOVA) with Tukey’s post hoc test was used for the comparison of the brandy composition. Dixon’s Q test was used for the detection of outliers in the data obtained (the analysis of each sample was performed in three parallels, the deviation of the five triplicate analyses was less than 5%).

## 3. Results and Discussion

### 3.1. Effect of Fermentation Type on the Analytical Profile of Plum Brandies

The fermentation of plum mashes with the addition of pure yeast culture was compared to fermentation with the autochthonous microflora of plums. Two batches of plum mash were inoculated with pure yeast cultures (SP7 and DV10) and one batch was fermented with natural autochthonous microflora. Analytical profiles and organoleptic properties of distillates produced from these ferments were compared. The results of the chemical analysis demonstrated significant differences between the spontaneously fermented sample (wild) and between the samples fermented with a pure yeast culture. The number of analytes detected in the distillates varied depending on the type of fermentation, with values ranging from 91 to 113. The sample of SP7 plum distillate exhibited the greatest number of detected substances. Table 1 presents a list of most abundant compounds. The significance of the differences between the plum varieties was assessed using ANOVA, with the results presented in Table 1 in the form of *p*-values. As illustrated in Table 1, a statistically significant disparity in analyte concentration was observed between the various fermentation processes (*p* < 0.05) for the majority of analytes. The brandy made from spontaneously fermented fruit (wild) differed from the samples inoculated with pure yeast by higher concentrations of acetaldehyde, 1-butanol, hexanol, nonanol, ethyl salicylate, α-terpineol and, conversely and, lower concentrations of methyl acetate, methanol, 2-butanol, 1-propanol, isobutyl alcohol, ethyl (-)-L-lactate, acetic acid, linalool, geraniol, benzyl alcohol, 2-phenylethanol, eugenol, allayl alcohol, ethyl octanoate, ethyl decanoate, ethyl benzoate, diethyl succinate, benzyl acetate, ethyl laurate, ethyl palmitate, ethyl stearate, ethyl oleate, ethyl linoleate, acetone, 2,3-butandione, and benzaldehyde. It can be stated that the spontaneously fermented plums exhibited a low content of esters (Table 1). Interestingly, the spirits made from spontaneously fermented fruit contained lower concentrations of ethyl esters of higher fatty acids. The formation of ethyl esters of fatty acids is associated with the formation of the yeast cell membrane [50], and their concentration increases with higher concentrations of yeast [2,6,50,51]. Pielech-Przybylská [52] observed that higher contents of acetic acid esters, particularly ethyl acetate and isoamyl acetate, and lower concentrations of fatty acid ethyl esters were achieved during spontaneous fermentation by epidermal microflora than in the case of fermentation led by inoculated yeast. Furthermore, the results of our study are consistent with this claim. Concurrently, the distillate produced by spontaneous fermentation exhibited a higher content of butanol (Table 1), which is reported to be a product of the metabolism of certain bacteria, such as *Clostridium* [53].

Significant differences from the other samples that could be found in the plum mash inoculated with SP7 yeast were mainly the significantly higher concentration of methylacetate, ethyl acetate, 2-butanol, 1-propanol, isobutyl alcohol, isoamyl alcohol, ethyl (-)-L-lactate, acetic acid, N-amylalcohol, linalool, octanol, decanol, geraniol, benzyl alcohol, 2–phenylethanol, eugenol, allyl alcohol, propyl acetate, isoamyl acetate, ethyl benzoate, diethyl succinate, benzyl acetate, and ethyl stearate. The plum brandy prepared by fermentation with culture SP7 had the lowest concentrations of acetaldehyde, acetone, furfural, and ethyl myristate (Table 1).

Brandy produced using SP7 yeast culture contained a relatively high concentration of allyl alcohol, which may indicate a more significant influence of bacterial microorganisms originating from the epiphytic microflora of the plums, whose metabolic products may have inhibitory effects on yeast [2,54]. The relatively higher content of ethyl acetate, isoamyl acetate, and benzyl acetate found in the plum spirit produced by SP7 yeast fermentation correlates well with the increased concentration of their precursor acetic acid. The elevated concentrations of these substances may also suggest a heightened metabolic activity of bacteria within the fruit fermentation [2].

The content of higher alcohols isobutyl alcohol, isoamyl alcohol, and 2-phenylethanol was the lowest in plum spirit made from spontaneously fermented fruit mash compared to other samples. This could be due to the different properties (enzyme production, nitrogen requirements, etc.) of wild and added “noble” yeasts. Satora and Tuszyński [7] report that non-*Saccharomyces* yeasts produce higher amounts of higher alcohols than wild yeasts, which is consistent with the measured results. Tsakiris [55] posits that the concentration of higher alcohols in distillates ranges between 250 and 500 g/hL 100% vol. ethanol. At a concentration of 350 g/hL 100% vol ethanol, higher alcohols have been reported to be indicative of sensory defects in plums [26,56]. Summing up the higher alcohols in our three plums gives values of 587, 1968, and 612 g/hL 100% vol ethanol for wild, SP7, and DV10, respectively. These are values that negatively affect sensory perception. It is estimated that 80–90% of this amount is involved in the concentration of 1-propanol, which is the most abundant higher alcohol in all three samples. The content of higher alcohols in fruit brandy is affected by the representation of microorganisms [31,52]. Apostolopolou et al. [57] state that the high value of 1-propanol is due to the development of bacterial cultures during fermentation, when air access occurs (in our case, this was not the case), or during the storage of the raw input material. Our results suggest that in the must inoculated with the SP7 yeast culture, the microbial culture was more developed than in the other two batches. It can be a challenge to adequately control the raw input material and store it for the shortest possible time on a large production scale where tens of tonnes of fruit are processed at a time. And as can be seen from the results for higher alcoholic strengths, the lowest concentration was for wild plum distillate, closely followed by the DV10 distillate. For the plum produced from SP7 ferment, the value was three times higher. This indicates that even the addition of a pure yeast culture at the beginning of the fermentation, if a bacterial microflora is developed in the raw material, does not guarantee an analytically and sensorially excellent distillate.

The plum brandy sample, fermented with the addition of yeast DV10, exhibited the highest methanol content among other samples, which could indicate the high pectinase activity of this yeast strain. The methanol content for distillate made from plums is regulated to a maximum of 12 g per litre of 100% vol. ethanol, according to regulation [17]. It follows that plum distillate DV10 would be hazardous to health if it were not mixed with other plum distillates in large quantities by the manufacturer. Furthermore, the sample contained relatively high concentrations of butyl acetate, ethyl benzoate, and ethyl esters of fatty acids, such as ethyl octanoate, ethyl decanoate, ethyl laurate, ethyl myristate, ethyl palmitate, and ethyl linoleate. Conversely, this sample exhibited the lowest concentrations of ethyl acetate, 1-butanol, N-amyl alcohol, hexanol, octanol, nonanol, decanol, α-terpineol, propyl acetate, isoamyl acetate, ethyl salicylate, and ethyl cinnamate (Table 1).

**Figure 2:**
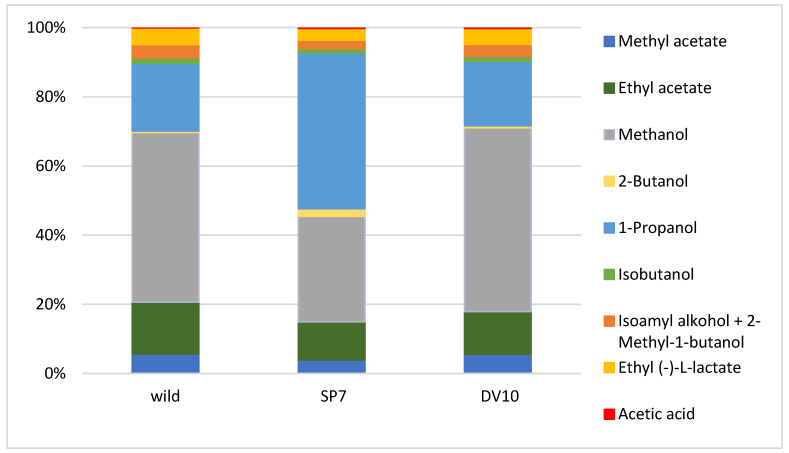
Mean relative contribution (%) of major compounds in plum brandies produced from wild, SP7, and DV10 yeasts fermentation.

**Figure 3:**
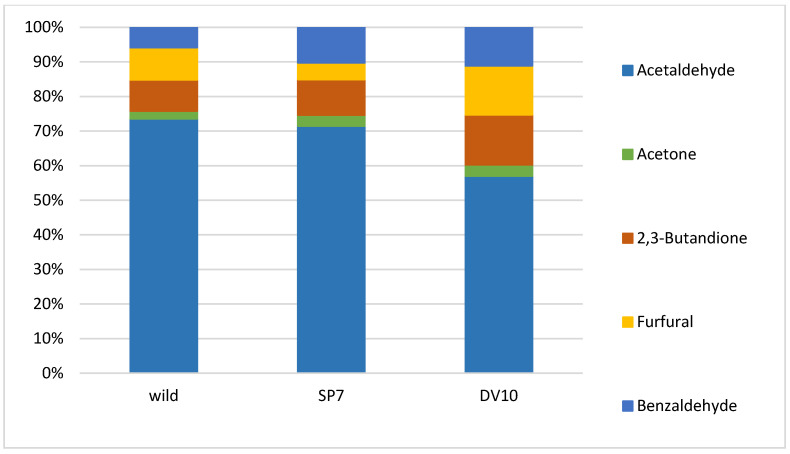
Mean relative contribution (%) of carbonyl compounds in plum brandies produced from wild, SP7, and DV10 yeasts fermentation.

**Figure 4:**
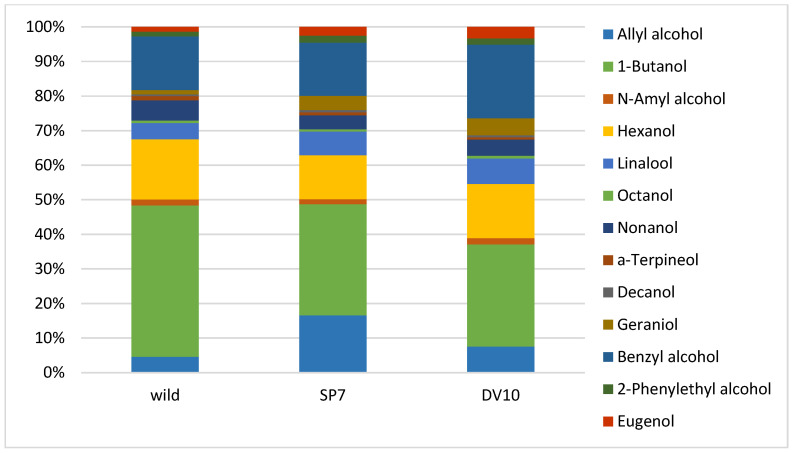
Mean relative contribution (%) of minor alcohols in plum brandies produced from wild, SP7, and DV10 yeasts fermentation.

**Figure 5:**
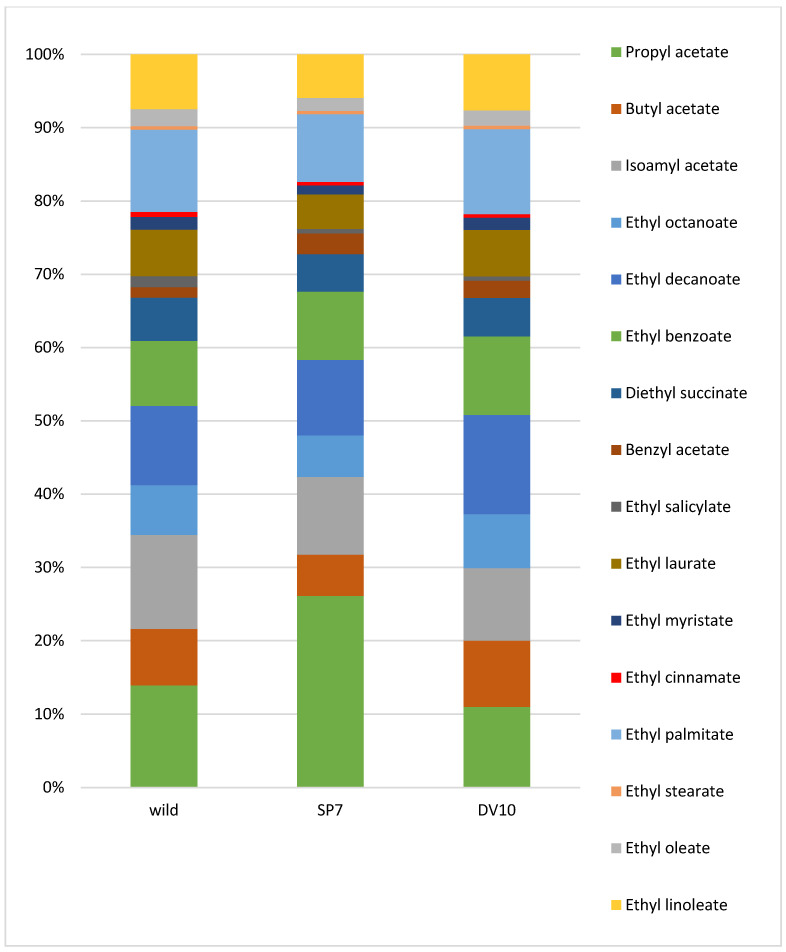
Mean relative contribution (%) of minor esters in plum brandies produced from wild, SP7, and DV10 yeasts fermentation.

Mean relative substances context (%) graphs of the analytical profile of final plum brandies were created to better represent the differences between individual groups of analytes (Figure 2, Figure 3, Figure 4 and Figure 5).

The distillates produced by pure culture fermentation exhibited a greater concentration of benzaldehyde than the plums derived from wild fermentation (Figure 3). Benzaldehyde is formed by the enzymatic conversion of its precursors in fruit stones during fermentation [6]. Benzaldehyde is also present in small amounts in the pulp of plums, where it can be converted to benzyl alcohol by yeast and, at low glucose concentrations, to benzoic acid [58,59]. Other substances that were produced in significant amounts in both distillates produced from plum mashes fermented with the addition of pure yeast culture were geraniol and linalool (Table 1). Plums do not contain these substances [26,60], but they are present in a glycosylated form [61]. The free form is only found in plums [30,62,63]. The transition to the non-glycosylated form is enzymatically influenced by temperature and pH 3, and, at the same time, the yeast forms or transforms terpenoids [60]. Thus, a high concentration of geraniol and linalool may indicate a high activity of yeast enzymes, such as glycosidases.

The relationships between the concentrations of substances in the different samples followed analogous formulae depending on their physico-chemical properties. Plum brandy made from fruit mash inoculated with yeast DV10 had the highest concentrations of most aldehydes, fatty acid esters, and, conversely, the lowest concentrations of many more volatile esters and alcohols. On the other hand, plum brandy produced from spontaneously fermenting fruit mash contained significantly lower concentrations of most of the less volatile compounds, including fatty acid esters [27,40,64]. Ethyl esters are the largest group of aromatic compounds in plum brandy. They are formed by yeast metabolism during fermentation and significantly influence the sensory character of the distillate [21]. Esters affect the overall profile of distillates more than higher alcohols [22].

The final plum brandy is produced by collecting the heart of the distillate during distillation over a relatively long period, while the composition of the distillate coming out of the column evolves over time. For this reason, samples were taken continuously at seven time intervals during distillation and the time course of the concentrations of the substances listed in Table 1 was monitored.

### 3.2. Changes in the Analytical Profiles of Plum Brandies during Distillation

The distillation process can have a significant impact on the analytical profile of the resulting distillate [65]. With the help of distillation, it is possible to mitigate some of the defects of the fruit mash, but its incorrect management can lead to the production of a distillate with undesirable organoleptic characteristics [66,67,68]. The organoleptic profile of the distillate is determined by a combination of primary (from the fruit), secondary (from fermentation), tertiary (from distillation), and quaternary (from ageing) aromatic compounds [7,21,69,70,71,72]. The compositions of the distillate obtained at the different stages of the distillation process are different. The evolution of the concentration of these substances depends on their physico-chemical properties and the composition of the distilled fruit mash. The analytical profiles of the distillates develop differently when different distillation techniques are used [4,21].

The development of analytical profiles of distillates during distillation was observed during the distillation of plums inoculated with SP7 and DV10 yeasts and spontaneously fermented plums (wild), using single pot still distillation (distillation using a distillation column). The evolution of the individual volatile concentrations during the distillation was carried out according to what is reported in the literature [6,27]. According to Léauté [27], the boiling point of the individual components and their miscibility with ethanol and water have the greatest influence on the development of concentrations of various substances during distillation. In our work, there is a significant difference in the evolution and concentration of some substances. Figure 5 shows the changes in the concentration of substances that reach their maximum at the beginning of the heart of the distillate. At the beginning of the distillation, the concentration of substances with low boiling points and good solubility in ethanol, such as methyl acetate, ethyl acetate, acetaldehyde, and acetone, decreased rapidly. The majority of acetaldehyde is separated in the head fraction [73], and its concentration decreased by 80% at 30 min of distillation of the heart fraction. As reported by Spaho et al. [21], ethyl acetate and isobutyl acetate are most abundant in the head fraction; therefore, their concentration in the heart fraction decreases significantly with the onset of the heart fraction. The concentrations of substances with higher boiling points, which are poorly miscible with water, well miscible with ethanol, and distilled with their vapours, are propyl acetate, butyl acetate, isobutyl acetate, ethyl butyrate, and isoamyl acetate, reaching maximum values at the end of the first half of the distillation [6].

**Figure 6:**
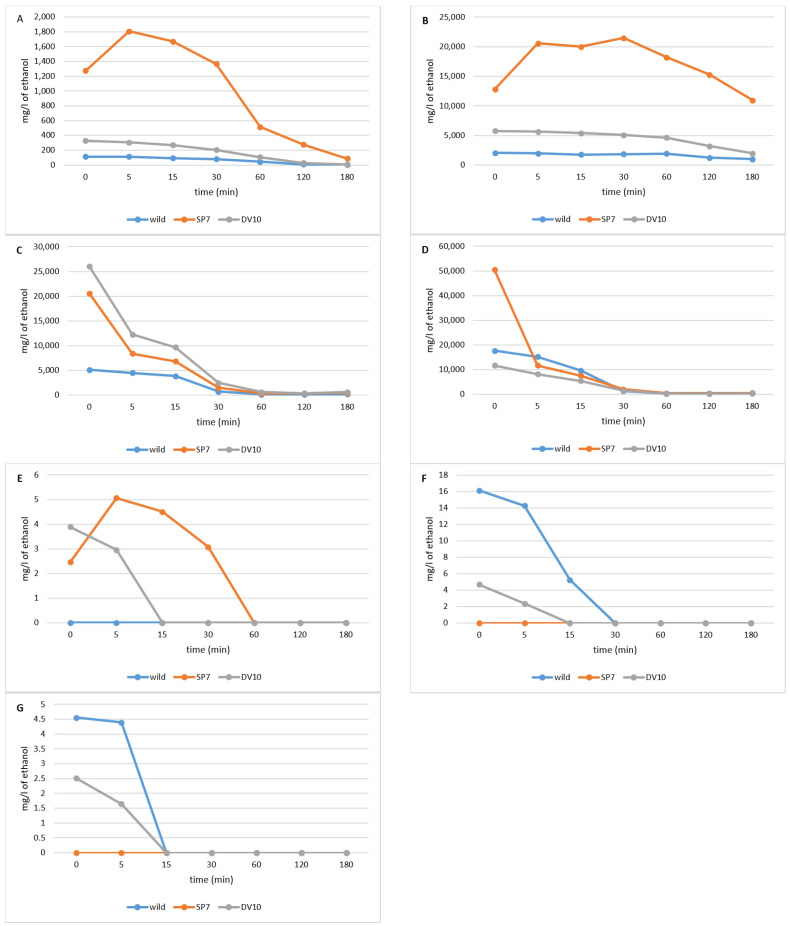
Evolution of the concentration of selected substances during the distillation of the heart fraction of plum distillates—decreasing concentration over time. (**A**)—2-butanol, (**B**)—1-propanol, (**C**)—methylacetate, (**D**)—ethyl acetate, (**E**)—nonanal, (**F**)—isobutyl acetate, (**G**)—hexyl acetate.

Compounds whose concentration increased significantly towards the end of the distillation (Figure 6) were methanol, furfural, linalool, α- terpineol, decanol, benzyl alcohol, eugenol, acetic acid, and ethyl lactate, similar to what was stated by Spaho et al. [21]. The formation of methanol by the demethylation of pectin occurs during fermentation but also during the entire storage period of fermented plum mash [10,11,12,13] until it is distilled [14]; therefore, fermented plum kvass should not be stored for a long time [15], but this is not always possible due to capacity/operational reasons.

The compounds which occurred in relatively high concentrations in the middle part of the distillation, or reached their maximum concentration here, were isobutyl alcohol, isoamyl alcohol, 2.3-butanedione, benzaldehyde, allyl alcohol. 1-butanol, hexanol, octanol, nonanol, ethyl benzoate, benzyl acetate, ethyl salicylate, and ethyl laurate. These compounds, which are well or partially soluble in ethanol and water, were eluted throughout the distillation period. According to what Spaho [6] states, the higher the boiling temperature, the higher the times at which they reached the highest concentrations. The concentrations of ethyl lactate, diethyl succinate, furfural, benzyl alcohol, 2-phenylethanol, eugenol, and acetic acid, which have high boiling points, were completely or at least partially soluble in water and carried away by its vapours as the end of the distillation approached and the relative proportions of water in the distillate and vapours increased [6,27]. The concentration of hydrophobic esters with high boiling points (such as ethyl esters of fatty acids) also increased, as they are likely to evaporate more freely with ethanol vapours as the polarity of the environment increases [6].

Significant differences are evident in the results between plum brandies produced using pure yeast cultures and those produced through spontaneous fermentation. The plum brandies obtained using yeast produced higher amounts of carbonyl compounds (nonanal, hexanal, benzaldehyde, and heptanal for brandy using DV10). Brandy using SP7 produced large amounts of allyl alcohol. The high concentration of allyl alcohol may indicate a more significant influence of bacterial microorganisms originating from the epiphytic microflora of the plums used to prepare this fruit mash, whose metabolic products may have inhibitory effects on yeast [2,54]. Distillates from pure cultures produced higher concentrations of esters than those from wild fermentation, in particular, butyl acetate, ethyl octanoate, ethyl decanoate, methyl benzoate, ethyl benzoate, benzyl acetate, 2-phenyl acetate, ethyl laurate, methyl palmitate, and ethyl palmitate. Wild fermentation produced more acetic acid, ethyl salicylate, hexyl acetate, and isobutyl acetate.

**Figure 7:**
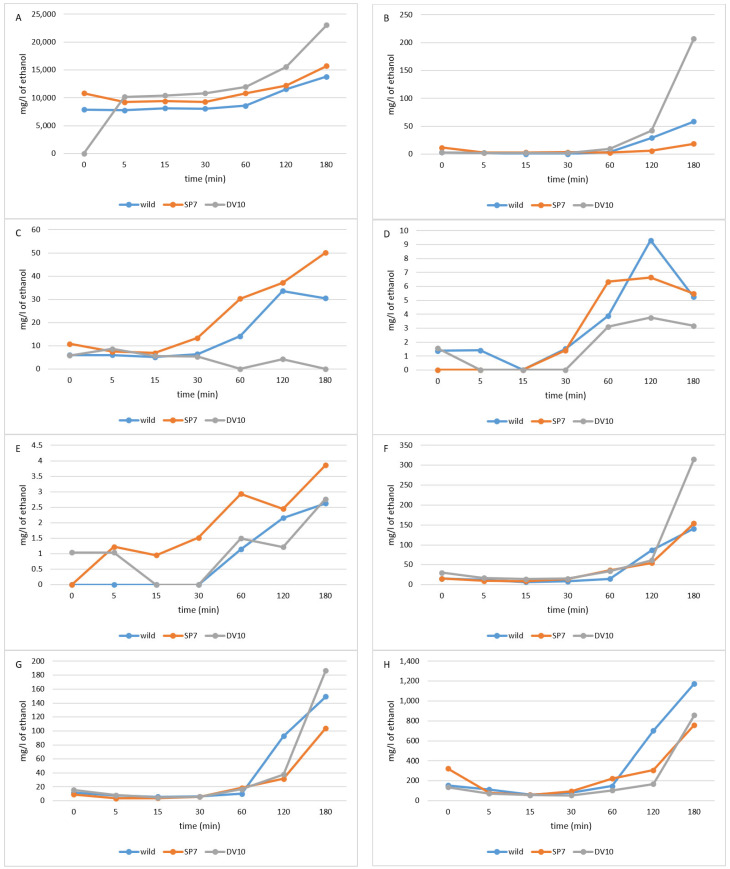
Evolution of the concentration of selected substances during the distillation of the heart fraction of plum distillates—increasing concentration over time. (**A**)—methanol, (**B**)—furfural, (**C**)—linalool, (**D**)—α-terpineol, (**E**)—dekanol, (**F**)—benzyl alcohol, (**G**)—eugenol, (**H**)—acetic acid.

It is noteworthy that there is a significant discrepancy between the SP7 and DV10 yeasts used in the concentration curves of certain substances. For example, the high content of 2-butanol and 1-propanol (Figure 6A,B) is striking for yeast SP7. The evolution of the concentration of the 1-propanol in the “heard” phase does not change significantly; as mentioned by Spaho et al. [21], it is not found in the head fraction, i.e., the beginning of the heart sampling does not affect the reduction in its concentration. Also, the concentrations of ethyl acetate (Figure 6D), nonanal (Figure 6E), linalool (Figure 6C), and decanol (Figure 7E) are significantly higher in plum brandy distilled from plums with the addition of pure-culture SP7. Plums inoculated with yeast DV10 led to significantly different concentration curves of methyl acetate (Figure 6C), methanol (Figure 7A), and furfural (Figure 7B). The wild fermentation plums had significantly different concentration curves of isobutyl acetate (Figure 6F), hexyl acetate (Figure 6G), and acetic acid (Figure 7H).

The initial and final fractions (heads and tails) of the distillate contain desirable and undesirable compounds from the perspective of organoleptic properties, and these fractions of the distillate are unsuitable for the production of fruit distillates [2,6]. The first phase is typically separated shortly after the start of distillation, at a time when the distillate has already lost most of its volatile compounds and has relatively mild organoleptic properties [21,40]. The separation of the final fraction, or the end of the collection of the heart of the distillate, is usually carried out when an alcohol content of 40–50% is reached [2,21]. In both monitored distillations, core sampling was completed when an alcohol content of approximately 45% (vol.) was reached. As mentioned by Spaho et al. [21], the ideal moment to stop removing the core of the distillate from the point of view of organoleptic properties is not fixed; it varies for the distillation of different kvass. This information is very important in the context of our results.

**Figure 8:**
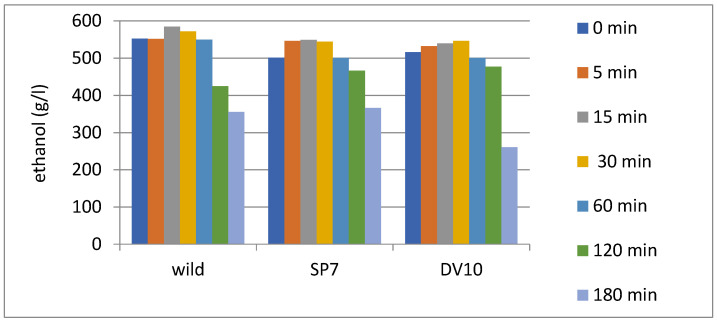
Evolution of ethanol concentration during the distillation of plum brandies.

From the point of view of the evolution of ethanol concentration during distillation in each fermentation type, ethanol reached higher concentrations during the distillation of spontaneously fermented plums (Figure 8). At the beginning of the distillation of plums inoculated with SP7 yeast, the ethanol concentration was lower compared to the distillation of spontaneously fermented plums and plums fermented with DV10 yeasts. It is interesting to note that the change in ethanol concentration in the distilled “heart” fraction was not statistically significant up to 120 min. Figure 8 shows that between 120 and 180 min, the ethanol content decreased from 43–47% to 27–37%. In terms of mean relative contribution, this represents only 10% of the volume of total ethanol. However, in terms of time, it is one-third of the total distillation time. At the same time, if we look at the substances that eluted mainly between 120 and 180 min (Figure 7), they were furfural, benzyl alcohol, eugenol, acetic acid, and also methanol. None of these substances are significant for the fruity or other positive character of plum brandies, rather the opposite. On the other hand, stopping the distillation after 120 min reduces the acetic acid content by 40 to 60% and the methanol content by 20 to 30%.

As already mentioned in the literature, methanol is a significant component of the heart fraction in the production of fruit distillates [40], and this tool that we present to reduce its content by 20% may therefore be of interest. This is especially so as some producers still prefer to ferment their mashes in open vats, where the resulting distillates often contain a methanol content above the legal limit, in addition to an increased amount of volatile acids [15]. Acetic acid content can also be effectively reduced by incorporating in-line conductivity measurement into the distillation process [42], although the possibility of modernising the distillation process is not always readily accepted by distillery operators (especially smaller ones). As stated by Popovic et al. [15], modification of the plum distillate production method contributes to the reduction in the content of undesirable components (methanol and acetic acid), and maintaining the optimal amount of higher alcohols, volatile acid esters, aldehydes, and benzaldehyde is highly desirable. In addition, the one-third reduction in gas consumption for heating and distilling of fruit mash is a significant positive result.

### 3.3. Sensory Analysis

The triangular test (Table 2), conducted with 39 assessors, showed that there were significant sensory perceptible differences between the “wild” sample and “SP7” sample, as well as between the “wild” sample and “DV10” sample (*p*-value lower than 0.001). Those assessors who were able to discern these differences were subsequently subjected to pairwise comparative testing with the respective samples. The preference for the SP7 sample was observed among the assessors compared to the wild sample (*n* = 32, *p*-value inferior to 0.001), while the preference was observed for the wild sample over the DV10 sample (*n* = 25, *p*-value inferior to 0.05).

**Figure 9:**
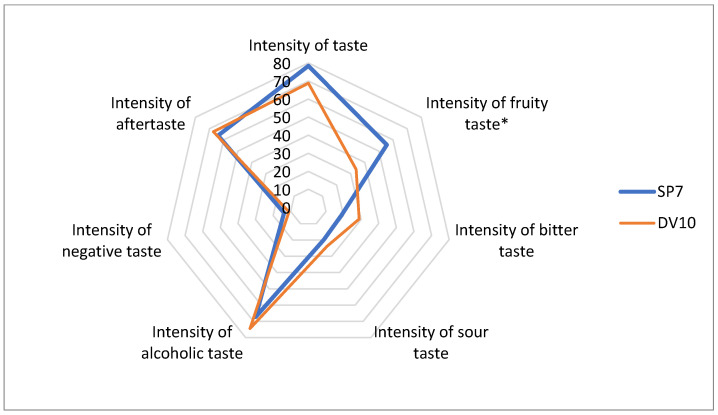
Comparison of taste profiles of samples fermented with the addition of pure yeast cultures. * Significant difference: *p* < 0.05.

**Figure 10:**
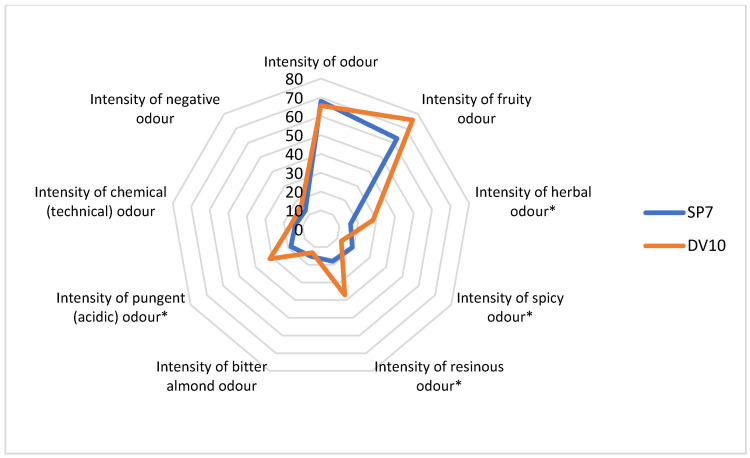
Comparison of odour profiles of samples fermented with the addition of pure yeast cultures. * Significant difference: *p* < 0.05.

**Figure 11:**
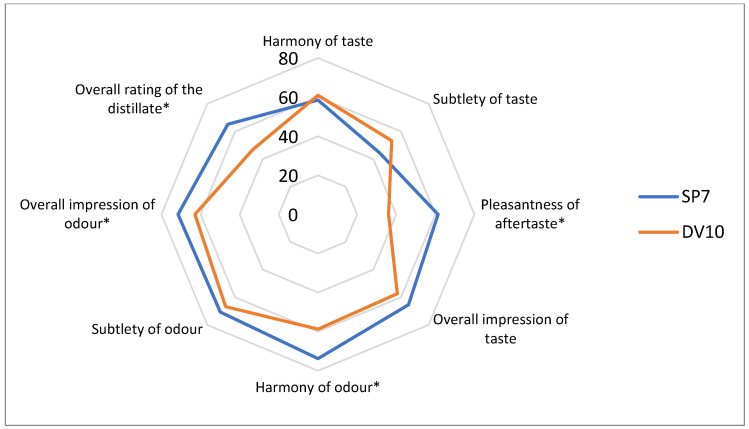
Comparison of additional sensory descriptors of samples fermented with the addition of pure yeast cultures. * Significant difference: *p* < 0.05.

During the descriptive analysis, statistically significant differences (*p*-value lower than 0.05) were observed for the following descriptors: intensity of fruity taste, harmony of odour, intensity of herbal odour, intensity of spicy odour, intensity of resinous odour, intensity of pungent (acidic) odour, overall impression of odour, overall rating of the distillate, and pleasantness of aftertaste (Figure 9, Figure 10 and Figure 11). Thus, it appears that the use of pure yeast cultures for fermentation leads to significant differences in the aroma and sensory perception of brandy. Although both pure yeast strains have very similar characteristics, including resistance to stress conditions (low pH), low consumption of nitrogenous substances, and a wide range of fermentation temperatures, they differ in sensory perception. This may be due to small differences in the enzyme production characteristics but also to the already mentioned probable difference in the storage of the input raw material and the delay in fermentation.

According to Ivanovic et al. [38], substances with positive sensory perception include amyl alcohols and 1-hexanol. 1-Hexanol has a perceivable sensory effect at a concentration greater than 20 mg/L and a negative effect at a concentration greater than 100 mg/L. Its concentration in all three of our plum brandies was in this range, as presented in Table 1, so it could have positively influenced organoleptic properties. The most common and typical ester of fruit distillate is ethyl acetate, which, at lower concentrations, adds floral notes to the beverage but in high concentrations brings solvent-like notes [74]. Differences in ethyl acetate content are probably the result of subtle differences in the composition of the bacterial and yeast microflora in the fermented mash [37]. Another ester found in higher quantities in the SP7 distillate is propyl acetate, which could also positively contribute to the organoleptic properties of the distillates. The most abundant aldehyde in spirits is acetaldehyde, which represents up to 90% of the carbonyl content [75]. If its concentration is less than 125 mg/L (which is in all our samples), it is a carrier of a pleasant fruity odour [32]. The distillate produced from DV10 ferment contained the highest concentrations of acetaldehyde, allyl alcohol, and 1-butanol. These substances adversely affect the distillate with their penetrating, pungent, and chemical odour [76,77]. Because they are volatile compounds that pass to the distillate during the initial phase of distillation, their higher concentration in this distillate can be attributed to the possibly earlier separation of the head fraction from the heart fraction. Our results suggest that due to the elevated content of these compounds in the DV10 distillate, this sample was not preferred compared to the wild sample. Cortés and Fernández [78] mentioned that the isobutanol/propanol ratio should be greater than 1. While the concentration of 1-propanol was several times higher than that of isobutanol in all three of our tested distillates, a negative sensory effect could be manifested here. Since the propanol content was significantly the highest in sample SP7 (and thus the isobutanol/propanol ratio was by far the lowest), which scored relatively positively in the sensory evaluation, this does not seem to have had a significant effect. Similarly, Satora and Tuszyński state that a concentration of propanol higher than the concentration of isobutanol does not affect the quality of the beverage [70].

Both distillates produced using pure yeast culture contained a higher proportion of higher alcohols (such as 1-propanol, isobutyl alcohol, and isoamyl alcohol) and ethyl esters of fatty acids than the “wild” sample. This phenomenon can be attributed to the activity of “noble” yeasts, particularly *Saccharomyces cerevisiae*, which is producing so-called “bouquet compounds”, which include higher alcohols, ethyl esters of fatty acids, and other compounds, which are contributing to the complex fruity and characteristic spirit-like organoleptic properties of the distillate [79]. Likely for this reason, the evaluators preferred these distillates over the “wild” distillate.

Furthermore, the sample of brandy produced from yeast inoculated with SP7 yeast contained high concentrations of carbonyl compounds, such as isovaleraldehyde, furfural, and benzaldehyde. The formation of isovaleraldehyde in fruit distillates is often associated with yeast activity, and its content in distillates produced using pure yeast cultures is in fact higher than in distillates from wild fermentation [52]. At certain concentrations, isovaleraldehyde contributes positively to the intensity of fruity aroma, but at excessively high concentrations, it can have a negative impact on the aroma and taste profile of the fruit distillate [80]. Furfural is formed during distillation by the thermal degradation of pentoses or by the Maillard reaction [81]. Low concentrations of this aldehyde can positively influence the sensory profile of fruit distillate with its bitter almond and cinnamon aroma. However, excessively high concentrations can be potentially toxic. A high concentration of furfural in fruit distillate may indicate a high content of fermentable pentoses in fruit mash [57]. Benzaldehyde is a key component of the almond aroma (and according to some studies, the raspberry aroma as well [4]) and may a possibly positively influence the olfactometry properties of fruit distillates [82]. Another substance found at higher levels in the SP7 distillate than in the DV10 distillate is propyl acetate, which, according to the above information, contributes to a pleasant fruity aroma.

## 4. Conclusions

In the Czech Republic and Germany alone, around 8 million litres of ethanol are produced annually from fruit fermentation. This includes both small and large industrial producers. The current scientific focus is on the investigation of product authenticity and the development of the most advanced analytical methods to identify and quantify as many substances as possible. However, the industry requires a different approach. The development of an accessible and cost-effective analytical method that can rapidly identify and quantify higher concentrations of undesirable substances is crucial. Such a method would enable the early detection of any potential problems, preventing any negative impact on sensory properties or the imposition of legislative restrictions on the volume produced. The results of our industry-led research indicate that the most critical step is to limit the storage of the raw input material, thereby reducing the likelihood of bacterial contamination and thereby reducing the subsequent risk of producing higher concentrations of 1-propanol. Furthermore, our results indicate that it is only possible to take the heart of the distillate up to an ethanol content of approximately 450 g/L. Distillation to lower concentrations results in an increase in the total distillate volume of about 10%. However, this is accompanied by a delay in the distillation time, which in turn leads to an increase in boiler heating costs of approximately 30%.

The distillates produced with the addition of pure cultures gave higher concentrations of esters than those obtained with wild fermentation, in particular butyl acetate, ethyl octanoate, ethyl decanoate, methyl benzoate, ethyl benzoate, benzyl acetate, 2-phenyl acetate, ethyl laurate, methyl palmitate, and ethyl palmitate. Wild fermentation resulted in higher concentrations of ethyl salicylate, 1-butanol, 1-hexanol, 1-nonanol, and α-terpineol.

## Figures and Tables

**Table 1:** Differences in the analyte concentration among the distillates produced with use of different plum fermentation methods determined as *p*-values (ANOVA test). * Significant difference: *p* < 0.05.

Major Compounds	RT (min)	Wild (mg/le)	SP7 (mg/le)	DV10 (mg/le)	*p*
Methyl acetate	4.81	1256	1500	1383	0.0144 *
Ethyl acetate	6.02	3354	4215	3078	0.0006 *
Methanol	6.31	10,954	11,715	13,188	0.0103 *
2-Butanol	11.5	105	834	149	4.74 × 10^−8^ *
1-Propanol	12.1	4395	17,287	4635	1.39 × 10^−7^ *
Isobutyl alcohol	14.3	330	456	357	0.0005 *
Isoamyl alcohol	18.4	850	932	855	0.1079
Ethyl (-)-L-lactate	22.7	1086	1318	1138	0.0070 *
Acetic acid	25.6	11.7	69.7	56.6	3.96 × 10^−7^ *
**Minor alcohols**					***p***
1-Butanol	16.2	118	108	70.8	6 × 10^−5^ *
N-Amylalcohol	19.2	4.85	5.03	4.16	0.0091 *
Hexanol	22.9	47.2	42.6	37.6	0.0043 *
Linalool	28.0	12.6	23.0	17.6	2.59 × 10^−5^ *
Octanol	28.3	1.93	2.03	1.78	0.055566
Nonanol	30.7	16.0	13.7	11.3	0.0005 *
Decanol	33.0	1.40	2.17	1.25	1.99 × 10^−5^ *
α-Terpineol	31.7	3.25	2.84	1.66	4.47 × 10^−5^ *
Geraniol	34.9	3.27	13.9	11.8	7.2 × 10^−7^ *
Benzyl alcohol	35.7	42.0	51.4	50.8	0.0054 *
2-Phenylethanol	36.4	3.63	6.91	4.36	1.06 × 10^−5^ *
Eugenol	41.41	3.76	8.47	7.98	6.09 × 10^−6^ *
Allyl alcohol	15.1	12.5	55.7	18.0	1.55 × 10^−7^ *
**Minor esters**	**RT (min)**	**wild (mg/le)**	**SP7 (mg/le)**	**DV10 (mg/le)**	***p***
Propyl acetate	9.36	34.8	91.0	33.2	5.29 × 10^−7^ *
Butyl acetate	13.5	19.1	19.5	27.2	0.0002 *
Isoamyl acetate	15.3	32.0	37.1	30.0	0.0050 *
Ethyl octanoate	25.1	16.9	19.7	22.2	0.0019 *
Ethyl decanoate	30.2	27.1	35.9	40.8	0.0002 *
Ethyl benzoate	31.1	22.1	32.5	32.3	0.0002 *
Diethyl succinate	31.2	14.8	17.9	15.9	0.0100 *
Ethyl salicylate	34.4	3.79	2.20	1.75	4.03 × 10^−6^ *
Benzyl acetate	32.5	3.55	9.77	7.11	2.42 × 10^−6^ *
Ethyl laurate	34.8	15.8	16.4	19.1	0.0070 *
Ethyl myristate	39.0	4.34	4.23	4.96	0.0151 *
Ethyl cinnamate	40.7	1.68	1.67	1.51	0.0724
Ethyl palmitate	42.8	28.0	32.2	35.0	0.0047 *
Ethyl stearate	46.3	1.24	1.57	1.41	0.0033 *
Ethyl oleate	46.7	5.76	6.21	6.28	0.1568
Ethyl linoleate	47.5	18.7	20.6	23.0	0.0061 *
**Carbonyl compounds**	**RT (min)**	**wild (mg/le)**	**SP7 (mg/le)**	**DV10 (mg/le)**	***p***
Acetaldehyde	3.54	105	91.4	93.4	0.0293 *
Acetone	4.61	3.21	4.02	5.25	6.75 × 10^−5^ *
2.3-Butandione	9.61	12.8	13.2	23.7	7.31 × 10^−6^ *
Furfural	26.1	13.3	6.16	23.2	6.11 × 10^−7^ *
Benzaldehyde	27.7	8.37	13.1	18.2	7.25 × 10^−6^ *

**Table 2:** Results of the sensory triangle test. * Significant difference: *p* < 0.05.

Samples	Number of Respondents	Correct Answers	*p*
SP7 vs. “wild” sample	39	32	<1 × 10^−6^ *
DV vs. “wild” sample	39	25	8 × 10^−5^ *

## Data Availability

The original contributions presented in the study are included in the article, further inquiries can be directed to the corresponding author.
